# The Prevalence of Labyrinthine Fistula in Chronic Otitis Media Surgery in Shiraz, Southern Iran

**Published:** 2011-08-01

**Authors:** A H Faramarzi, S T Heydari, M Rusta

**Affiliations:** 1 Depatrment of Otolaryngoloy, Shiraz University of Medical Sciences, Shiraz, Iran; 2 Depatrment of Biostatistics, Shiraz University of Medical Sciences, Shiraz, Iran

**Keywords:** Labyrinthine fistula, Chronic otitis media, Tympanomastoidectomy, Iran

## Abstract

**Background:**

The incidence of fistulas found during the surgery for chronic otitis media with cholesteatoma has been reported in a wide range in different geographical areas. This study aims to find the prevalence of labyrinthine fistula in the south of Iran.

**Methods:**

A prospective cross sectional study of 787 (504 ears belong to 462 patients) consecutive tympanoplasty with or without mastoidectomy for chronic otitis media was performed. Data on preoperative clinical and preoperative and postoperative hearing status and intraoperative findings were analyzed.

**Results:**

A labyrinthine fistula was found at surgery in 24 (4.7%) ears of the total 504 ears belonging to 462 patients undergoing surgery for chronic otitis media. Location of the fistula was the lateral semicircular canal in 23, posterior semicircular canal in 1 and promontory in 1 subject. There was a statistically significant difference in preoperative and postoperative AC threshold in 500-3000 Hz frequency and ABG 500-3000 Hz, but there was no statistically significant difference in the other variables.

**Conclusion:**

Regarding postoperative hearing outcome in the labyrinthine fistula surgery, it seems that there is no universal method of reporting of hearing results. Past hearing evaluation methods in the literature have been often poorly comparable, based on different methodology.

## Introduction

Labyrinthine fistulae are caused by abnormal communications between the inner ear and surrounding structures.[1]Bone resorption of the otic capsule is generally a consequence of longstanding cholesteatomatous otitis media and only in rare cases or in postmortem studies has been associated with inflammatory ear abnormalities other than cholesteatoma.[2][3] The incidence of fistulae during the surgery for chronic otitis media together with cholesteatoma has been reported in a wide range in different geographical areas and countries ranging between 2.9% and 12.5%.[4]The management and surgical technique, in turn, is the most controversial topic in the literature.[5]Because of above controversies, we designed this study to find the rate of the incidence of labyrinthine fistula in the south of Iran; with hope of filling some gaps pertaining to these controversies.

## Patients and Methods

This is a cross sectional study of 787 (504 ears belong to 462 patients) consecutive tympanoplasty with or without mastoidectomy for chronic otitis media. The operations were performed at Dastghaib Hospital affiliated to Shiraz University of Medical Sciences as a large otologic referral center in the south of Iran between 2003 and 2008. The preoperative clinical data included the patient's gender and age, presence of vertigo, preoperative (one week before surgery) and postoperative (3 months after surgery) hearing status, symptoms and physical findings were recorded in a question- naire. Chi Square and paired t test were performed for finding out the association between variables.

## Results

There were a total of 787 ear surgeries on 462 patients. Two hundred and forty (52%) females and 222 (48%) males patients were enrolled. Forty six (9.95%) patients were in pediatric (7-15 years) and 416 (90.04%) were in adult age groups. There was no statistically significant difference on incidence of labyrinthine fistula between pediatric and adult groups. A labyrinthine fistula was found at surgery in 24 (4.7%) ears belonging to 462 patients undergoing surgery for chronic otitis media. From 504 ears, there were two cases of blue line lateral semicircular canal (0.4%). Fistula test was positive in 12 out of 24 patients (50%). Frequency of the clinical signs and symptoms of the 24 patients with labyrinthine fistula was subjective hearing loss (24 patients), otorrhea (19 patients), otalgia (16 patients), tinnitus (15 patients) and vertigo (10 patients), respectively. Of the 24 cases with labyrinthine fistulas, cholesteatoma was found in the mastoid or middle ear in 21 ears (87.5%), and both cholesteatoma and granulation tissue in 3 ears (12.5%).

Location of the fistula was the lateral semicircular canal in 23, posterior semicircular canal in 1, and promontory in 1.Two ears had more than one fistula, both of them had fistulas in the lateral semicircular canal and posterior semicircular canal.There were 6 cases of labyrinthin fistula with a size of less than 2 mm, and 19 cases with a size of 2-4 mm. Also none of these fistulas were > 4 mm.There were 5(20.83%) deaf ears preoperatively. These preoperative deaf ears were omitted form hearing evaluation, so hearing evaluation in 19 cases with labyrinthine fistula was done. There were 11 (57.9%) ears with conductive and 8 (42.1%) ears with mixed hearing losses. Anakusis did not occur postoperatively, and a hearing improvement of more than 10 dB in SRT occurred in 4 (20.0%) ears.

The fistulae were sealed with a cortical bone chip and bone wax (8 patients, 33.3%), temporalis fascia and bone wax (8 patients, 33.3%), cortical bone chip and temporalis fascia (5 patients, 20.8%) and cortical bone chip, temporalis fascia and bone wax (3 patients, 12.5%).

There was a statistically significant difference in preoperative and postoperative AC threshold in 500- 3000 Hz frequency and ABG 500-3000 Hz (P<0.05), but there was no statistically significant difference in other variables (Table 1). There was 15 (62.5%) cases of facial nerve dehiscence in patients with labyrinthine fistula while 61 (12.7%) in other patients (OR =11.4, p<0.001).

**Table 1:** The preoperative and postoperative hearing status in patients with abyrinthine fistula

****	**No.**	**Mean **	**Standard deviation**	**P value**
SDS pre	19	15.8	87.3	0.078
SDS post	19	17.8	80.8	
SRT pre	19	16.8	56.8	0.073
SRT post	19	14.0	52.4	
BC 500-3000 pre	19	17.3	22.9	0.919
BC 500-3000 post	19	18.2	22.6	
AC 500-3000 pre	19	20.5	62.1	0.035
AC 500-3000 post	19	17.8	54.7	
BC 4000 pre	19	28.0	32.6	0.293
BC 4000 post	19	24.1	28.4	
AC 4000 pre	19	25.5	66.8	0.226
AC 4000 post	19	23.9	60.5	
ABG 500 pre	19	17.3	42.9	0.039
ABG 500 post	19	18.5	32.1	
ABG 1000 pre	19	16.5	42.1	0.016
ABG 1000 post	19	15.9	28.9	
ABG 2000 pre	19	14.8	36.1	0.040
ABG 2000 post	19	13.9	26.1	
ABG 3000 pre	19	14.0	36.8	0.043
ABG 3000 post	19	14.2	28.7	
ABG 4000 pre	19	13.0	36.3	0.298
ABG 4000 post	19	16.8	31.3	

## Discussion

The incidence of labyrinthine fistula was 4.67% in our study and it is within the range of the incidence of labyrinthine fistula in the literatures. Overall, the incidence of labyrinthine fistulas secondary to chronic otitis media in the modern literature varies from 3 to 13 percent.[3][4][5][6][7][8][9]Hakuba et al. recently reviewed 375 revision surgeries performed for recurrent chronic otitis media. Labyrinthine fistulae were recognized at revision surgery in 29 ears (7.73%).[1][6] The difference between our results and that of Hakuba et al.'s study is that all ear surgeries in Hakuba et al.'s study were recurrent chronic otitis media, but in the present study were revision surgeries for a second look.

Location of the fistula was the lateral semicircular canal (95.83%) and in Grewal et al.'s study was the lateral semicircular canal (96%).[7] Most studies have reported the incidence of isolated lateral canal fistulas to be about 80%, but this figure ranges from 57 to 91 percent.[3][4][6][8] Their findings are somehow similar to our findings.

In our service, we normally conducted canal wall-down mastoidectomy in cases of cholesteatoma with labyrinth involvement owing to the socioeconomic characteristics of the population, having a high prevalence of cholesteatoma of large extension at the first diagnosis.Our method of surgery is similar to that of Manolidis,4 Greenberg and Manolidis[8] Grewal et al.[7] and Penido and coworkers.[10]

Other studies, however, reported a high incidence of facial nerve dehiscence in fistula cases, (27-55%).[5] The higher incidence of facial nerve dehiscence in fistula cases in our study is probably due to the fact that in Iran, more cases of unsafe chronic suppurrative otitis media with more extensive cholesteatoma are seen and also most of our patients presented very late due to poor socioeconomic conditions.

The average preoperative deafness was 8.5%, the average surgically (surgical manipulation) related deafness 10%, and the total deafness rate due to the fistula was 18.5% in a few studies.[4][8] In a review article, 85% were reported as having unchanged or improved hearing postoperatively. Eight percent of patients experienced decreased hearing postoperatively. Six percent of patients with labyrinthine fistula experienced a dead ear postoperatively.[3] Although postoperative hearing outcome was similar to that of several above mentioned articles, none of our patients experienced deafness as a result of surgical manipulation. Regarding postoperative hearing outcome in labyrinthine fistula repair, past hearing evaluation methods have been often poorly comparable, based on different methodology. The limitation of this study was that CT scan of the temporal bone was not done preoperatively in most of patients. Although the need for preoperative imaging for cholesteatoma cases is debating, recent studies reported CT scans to be no more sensitive than history, physical examination, and clinical suspicion when making a decision to explore for labyrinthine fistulae Because of relatively, low incidence of labyrinthine fistula, we suggest future long term, multi-central studies, but with the same audiometric measures to obtain prospective data for exploring and estimating differences between methods of repair for labyrinthine fistula and other aspects in this filed.
